# The COVID‐19 world – Are we there yet?

**DOI:** 10.1111/jdi.13605

**Published:** 2021-07-08

**Authors:** Yoshiyuki Hamamoto

**Affiliations:** ^1^ Yutaka Seino Distinguished Center for Diabetes Research Kansai Electric Power Medical Research Institute Kobe Japan; ^2^ Center for Diabetes, Endocrinology and Metabolism Kansai Electric Power Hospital Osaka Japan

## Abstract

The spreading of SARS‐CoV‐2 virus infection is still of great concern as well as clinical and social interest. The key to conquer this pandemic would be establishment of herd immunity by vaccination and of treatment, and I have discussed issues we are facing now.

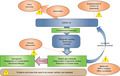

The emergence of variants of respiratory syndrome coronavirus 2 (SARS‐CoV‐2) and their rapid spread over the world has cast a shadow over potential victory enabled by novel and highly effective vaccines over this human tragedy. Coronavirus disease 2019 (COVID‐19) cases surpassed 100 million on January 27, 2021 according to the Center for Systems Science and Engineering at Johns Hopkins University, indicating that more than 1 in 80 humans on earth have already been infected. Some of the variants, especially the UK coronavirus variant (B.1.1.7), the South Africa variant (B.1.351), and the recently identified India variant (B.1.617), are more infectious and deadlier compared with SARS‐CoV‐2 before their appearance. The currently known major variants are characterized by mutations in the receptor‐binding domain of their spike‐protein (e.g., N501Y, E484K, and K417T/N); the India variant has two characteristic mutations of E484Q and L452R that may impact vaccine efficacy. COVID‐19 is distinguished from other infectious diseases by its two‐faced clinical presentation: it is sometimes almost asymptomatic despite its transmission potential, or it may present with pneumonia that can lead to rapid disease progression and a fatal outcome.

As diabetes and obesity are well known risk factors for the severity and mortality of COVID‐19, maintaining good glycemic control and appropriate body weight during the pandemic has become ever more important for patients and their physicians. While healthy eating and adequate physical activity are essential for patients with diabetes to maintain their glycemic control and body weight at all times, it has been reported that the containment measures for COVID‐19 negatively affect both the nutrition and exercise of patients with diabetes. We have reported that even under the mild containment measures imposed in Japan, patients with diabetes exhibited lifestyles altered toward a more sedentary mode, which caused a deterioration of glycemic control and weight gain, more seriously in the elderly population^1^. Even slightly reduced regular daily activities in elderly patients can eventually lead to a loss of skeletal muscle mass, and could pose a serious social health issue in the future. Thus, maintaining the physical activity level under a stay‐at‐home order is especially important for maintaining muscle mass in the elderly (Figure 1).

**Figure 1|:**
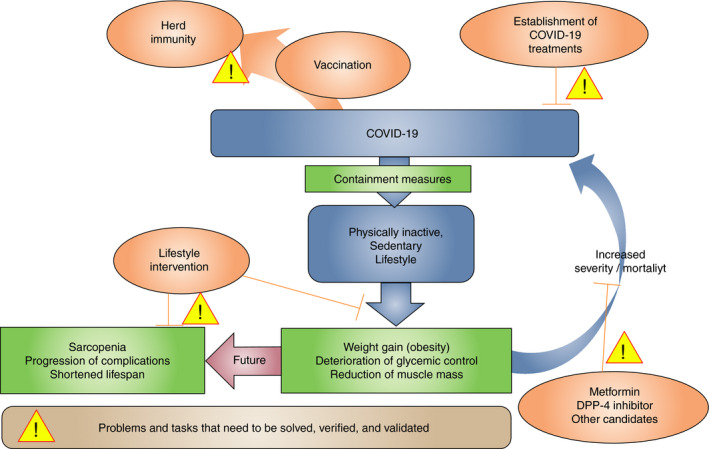
The schematic illustration of the problems and tasks that need to be solved, verified, and validated to conquer COVID‐19 and related issues in diabetes.

Treatment of the disease and establishment of herd immunity are necessary to conquer the pandemic. Special interest is focused on the efficacy of currently available vaccines, most of which employ novel messenger ribonucleic acid and virus vector technologies, against the emerging variants of SARS‐CoV‐2. mRNA vaccines are created synthetically using a fragment of the RNA sequence of the virus surface protein and are taken up by muscle cells at the injection site with the possible addition of fibroblast and dendritic cells; the synthesized virus protein confers the subsequent immune response. The expected effect of vaccines is not only the prevention of infection but also the reduction or prevention of transmission of SARS‐CoV‐2 including variants.

Vaccination not only protects against symptomatic illness but also mitigates the more severe manifestations of the disease, as well as the mortality rate. The mRNA vaccines show over 90% efficacy in preventing both symptomatic illness and severe disease of COVID‐19 in randomized clinical trials; in a real‐world setting, 7 days after the second dose, the Pfizer‐BioNTech mRNA vaccine showed over 90% efficacy for documented infections, symptomatic illness, and severe disease (hospitalization was 87%) in an analysis based on Israel’s mass‐vaccination^2^. In general, the Pfizer‐BioNTech vaccine and Moderna vaccine show sufficient protection against the B.1.1.7 and B.1.351 variants, although concern for efficacy against the B. 1.351 variant was raised in some reports; the efficacy against B.1.617 remains unknown. Furthermore, considering the recently published results of the reduced neutralizing activity of plasma collected from vaccinated volunteers against SARS‐CoV‐2 carrying the K417N, K417T, E484K, and/or N501Y mutations^3^, the efficacy of the vaccines on these and future variants is not yet known.

It is not known whether the vaccines are similarly effective among patients having underlying diseases, especially those associated with an immunocompromised state such as diabetes, cancer, and immune disease. We found that in elderly Japanese patients with diabetes, although a single vaccination of Oka varicella zoster vaccine safely and successfully induced a humoral and cell‐mediated immune response, concurrent vaccination of Oka varicella zoster vaccine and 23‐valent pneumococcal polysaccharide vaccine (PPSV23) induced a reduced immune response to varicella zoster vaccine^4^. Interestingly, we found that users of dipeptidyl peptidase 4 (DPP‐4) inhibitors show an attenuated immune response to the vaccine. This raises a concern about the efficacy of COVID‐19 vaccines in patients with diabetes who are taking certain antidiabetic drugs or/and immunosuppressive drugs. This issue requires further investigation.

Regarding treatments, while there is no magic bullet, corticosteroids such as dexamethasone are established drugs for the amelioration of the cytokine storm sometimes seen in patients with COVID in intensive care units (ICU). A Janus kinase inhibitor, baricitinib, is recently drawing attention as an alternative anti‐inflammatory drug. Currently, the authorities recommend only limited drugs including the anti‐viral drug remdesivir, dexamethasone, and tocilizumab optionally for hospitalized patients in a severe state. Baricitinib is recommended only when corticosteroids cannot be used in combination with remdesivir; there is not enough evidence to justify the first line use of baricitinib. The results of an ongoing clinical trial, Adaptive COVID‐19 Treatment Trial 4 (ACTT‐4), evaluating the drug’s effects against dexamethasone is awaited. The interleukin‐6 (IL‐6) inhibitor, tocilizumab, a recombinant humanized monoclonal antibody against IL‐6 receptors, was reported to protect from fatality when used in addition to standard care such as corticosteroids and anti‐viral drugs such as remdesivir in patients with severe or critical COVID‐19 infection and elevated inflammatory markers. This finding was presented in the recent results from the open‐label, pragmatic Randomized Evaluation of COVID‐19 Therapy (RECOVERY) trial^5^ as well as the Randomized, Embedded, Multifactorial Adaptive Platform Trial for Community‐Acquired Pneumonia (REMAP‐CAP)^6^. Although there is still controversy about the effectiveness of tocilizumab, a regular effect can be expected in certain clinical conditions.

Early diagnosis and the initiation of treatment as soon as possible are key to overcoming COVID‐19 infection. Recently, monoclonal antibodies developed for SARS‐CoV‐2 (to be used as combination therapy such as bamlanivimab and etesevimab or casirivimab and imdevimab) are being used in the treatment of patients with mild to moderate COVID‐19 who are not hospitalized but at high risk of disease progression and/or hospitalization, for example patients with diabetes, chronic kidney disease, and severe obesity. They are available only in limited countries and their application is restricted due to their administration by infusion. In this respect, the development of molnupiravir, an orally administered bioavailable prodrug of the ribonucleoside analog β‐d‐N4‐hydroxycytidine, is expected and is currently under phase 3 clinical trial for outpatients with COVID‐19.

Glycemic control before and during COVID‐19 infection in addition to the drugs used for hypertension and diabetes treatment are also significant factors affecting the severity and mortality of the disease. Glycemic control is an essential strategy for COVID‐19 treatment; poor glycemic control results in impaired immune defense and an abnormal immune response, which in COVID‐19 infection can lead to severe pneumonia and acute respiratory distress syndrome^7^. It is also thought that impairment of vascular endothelial cells due to poor glycemic control is involved in the poor outcome of patients with diabetes in the progression of COVID‐19 infection. There is both expectation and concern regarding glucose‐lowering drugs in relation to COVID‐19 infection. As suggested in the Sitagliptin Treatment in Diabetic COVID‐19 Positive Patients (SIDIACO‐RETRÒ) study reported by Italian researchers, DPP‐4 inhibitors may reduce mortality in patients with COVID‐19 infection^8^. The effect was confirmed by a meta‐analysis study^9^. Metformin is also expected to have a beneficial effect in terms of reducing mortality due to COVID‐19 infection, despite the concern for increased lactic acidosis by a hazard ratio of 4.66 (95% confidence interval: 1.45–14.99) as summarized in the article by Lui *et al*.^10^. The relation between glucose‐lowering drugs and the severity and mortality of COVID‐19 infection in diabetes patients is still largely unknown and warrants further investigation.

Again, the key to the successful treatment of severe COVID‐19 infection is early diagnosis and prompt initiation of treatment. In this respect, patients with diabetes should have better access to medical services than they do presently. Moreover, if herd immunity and effective treatment are not established, those who are at highest risk from infection of COVID‐19 would have to continue in voluntary lock‐down indefinitely. The efficacies of the vaccines and treatments in certain groups such as patients with underlying diseases including diabetes, require verification.

## Disclosure

Y Hamamoto received speaker fees from Novo Nordisk Pharma Ltd and received a research grant from Sumitomo‐Dainippon Pharma Co., Ltd and Nippon Boehringer Ingelheim.
